# Knockdown of liver cancer cell‐secreted exosomal PSMA5 controls macrophage polarization to restrain cancer progression by blocking JAK2/STAT3 signaling

**DOI:** 10.1002/iid3.1146

**Published:** 2024-02-02

**Authors:** Shujie Xie, Xiang Li, Jia Yan, Hua Yu, Shuhuai Chen, Kana Chen

**Affiliations:** ^1^ Department of Hepatobiliary and Pancreatic Surgery Ningbo No.2 Hospital Ningbo Zhejiang China; ^2^ Department of Plastic Surgery Ningbo No.2 Hospital Ningbo Zhejiang China

**Keywords:** exosomes, hepatocellular carcinoma, JAK2/STAT3 signaling, M2 macrophage, PSMA5

## Abstract

**Introduction:**

Tumor‐associated macrophages, a major component of the tumor microenvironment, undergo polarization into M2 macrophages (M2), and thereby exert an immunosuppressive effect to induce cancer metastasis. This study strives to uncover a molecular mechanism underlying this event in hepatocellular carcinoma (HCC).

**Methods:**

Proteasome subunit alpha 5 (PSMA5) expression in liver hepatocellular carcinoma (LIHC) tissues and its association with LIHC patients were predicted using StarBase. PSMA5 level in human HCC cells was manipulated via transfection. Exosomes were isolated from HCC cells, and internalized into macrophages which were cocultured with HCC cells. Exosome internalization was observed after fluorescence labeling. HCC cell migration and invasion were evaluated by wound healing and Transwell assays. Xenograft assay was performed to investigate the role of PSMA5 in in vitro tumorigenesis. M2 polarization was assessed by enzyme‐linked immunosorbent assay, quantitative reverse transcription polymerase chain reaction, and immunohistochemistry. PSMA5 expression in exosomes and Janus Kinase 2 (JAK2)/signal transducer and activator of transcription 3 (STAT3) activation in macrophages and tumors were detected by Western blot analysis.

**Results:**

High PSMA5 expression was observed in LIHC tissues and associated with compromised survival of LIHC patients. PSMA5 knockdown inhibited HCC cell migration and invasion. PSMA5 knockdown in HCC cells downregulated PSMA5 level in exosomes from these HCC cells. HCC cell‐isolated exosomes were successfully internalized into macrophages, and facilitated M2 polarization and JAK2/STAT3 pathway activation. HCC cell‐secreted exosomal PSMA5 knockdown inhibited the exosome‐induced effect on macrophages, and attenuated the promotion induced by exosome‐treated macrophages on HCC cell migration/invasion and tumorigenesis along with in vivo M2 polarization and JAK2/STAT3 pathway activation.

**Conclusion:**

HCC cell‐secreted exosomal PSMA5 knockdown hinders M2 polarization to suppress cancer progression by restraining JAK2/STAT3 signaling.

## Graphical abstract

High proteasome subunit alpha 5 (PSMA5) expression was observed in liver hepatocellular carcinoma (LIHC) tissues and associated with compromised survival of LIHC patients. PSMA5 knockdown inhibited migration and invasion of hepatocellular carcinoma (HCC) cells. PSMA5 knockdown in HCC cells downregulated PSMA5 in exosomes from these HCC cells.

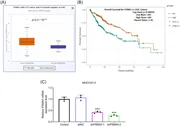

## 1 INTRODUCTION

Hepatocellular carcinoma (HCC) is a common malignancy arising from hepatocytes, leading to extremely bleak outcomes worldwide.^1^ HCC can be treatable with locoregional therapy at early stages; however, low sensitivity of currently utilized biomarkers delays the diagnosis, which favors cancer progression into the advanced or metastatic stage where the tumor become unresectable and only can be treated with systemic therapy such as the multikinase inhibitors, with a limited therapeutic effect and high likelihood of drug resistance.^1^, ^2^, ^3^ Hence, preventing the progression of HCC into the advanced stage is the key to bring a favorable outcome of patients.

It is generally believed that chronic inflammation contributes to the occurrence and progression of HCC.^4^ The recruitment and infiltration of tumor‐associated macrophages (TAMs) into the tumor microenvironment (TME)^5^ are associated with the poor prognosis of HCC patients.^6^ Once TAMs are recruited in the TME, they will undergo a dynamic change and polarize into two extremes, classically activated proinflammatory macrophages (M1) with antitumor properties, and alternatively activated anti‐inflammatory macrophages (M2) with pro‐tumor properties.^7^ During HCC progression throughout the early stage to the metastatic stage, TAMs tend to switch from M1 to M2.^8^ M2 has been reported to secrete growth factors, cytokines, and extracellular matrix components that support tumor cell survival, angiogenesis, and metastasis, and contribute to immunosuppression.^9^, ^10^, ^11^ Given the essential role of TAMs in tumor progression, targeting TAMs has emerged as a potential therapeutic strategy. The relative approaches have been explored in preclinical studies.^12^, ^13^ However, more research is needed to determine the efficacy and safety of these strategies in clinical settings.

Exosomes are a class of 50−150 nm‐diameter‐long, cup‐shaped extracellular vesicles that release from the inward budding of the plasma membrane in nearly all mammalian cells including cancer cells.^14^ Exosomes are rich in multiple receptors, proteins, microRNAs, mRNAs, DNAs, and metabolites.^15^ Through transportation of these molecules into recipient cells, exosomes function critically in mediating intercellular communication.^16^ On one hand, HCC cell‐derived exosomes contribute to the inflammatory response within the TME by carrying proinflammatory factors, including cytokines (such as interleukin [IL]‐6, IL‐8) and chemokines, which promote inflammation and recruit immune cells.^17^ On the other hand, HCC cell‐derived exosomes transfer various bioactive molecules to M2 infiltrating the TME, thereby promoting HCC immune escape.^18^ Additionally, HCC cell‐derived exosomes can transfer genetic material, such as miR‐452‐5p, which can regulate gene expression in recipient cells and potentially affect immune and inflammatory pathways.^19^ On account of these findings, we speculated that HCC cell‐derived exosomal protein has a role in modulating HCC tumorigenesis by driving TAM polarization into M2.

The proteasome subunit alpha (PSMA) protein family, which is essential to the assembly of the 20S proteasome core complex,^20^, ^21^ has been reported to be enriched in exosomes derived from highly metastatic HCC cells.^22^ PSMA5, a member of the PSMA protein family, is implicated in many cancerous diseases.^23^ Previously, aberrantly high expression of PSMA5 has been detected in prostate cancer,^24^ lung adenocarcinoma (LUAD),^25^ pulmonary neuroendocrine tumors,^26^ and endometrial cancer,^27^ hinting its associations with progression of above cancers.^25^ However, PSMA5 expression in HCC and its association with HCC progression remain unclear. Noteworthily, PSMA5 is lowly expressed in TAMs showing M1‐like phenotype,^28^ and is capable of activating the Janus kinase 2 (JAK2)/signal transducer and activator of transcription 3 (STAT3) pathway,^25^ which has emerged as an important path involved in the polarization into M2.^29^ These findings combined with the notion that M2 is tumorigenic collectively suggest that PSMA5 may favor M2 polarization by activating the JAK2/STAT3 pathway, thus promoting HCC progression. Since PSMA5 is detected in HCC cell‐derived exosomes, we further hypothesized that PSMA5 may be a key protein that is transferred by HCC cell‐derived exosomes to TAMs and thus drives M2 polarization to propel HCC progression.

The present study is committed to investigating whether the involvement of PSMA5 in HCC progression can be attributed to its facilitation of M2 polarization through exosomal release from HCC cells. By exploring this novel mechanism of PSMA5 as a key regulator of HCC metastasis, we aim to shed light on the broader understanding of HCC pathogenesis and potentially reveal the exosomal PSMA5 as a promising target for HCC treatment.

## 2 METHODS AND MATERIALS

### 2.1 Bioinformatics analyses

StarBase (https://starbase.sysu.edu.cn) was utilized to predict the expression pattern of PSMA5 in liver hepatocellular carcinoma (LIHC) tissues, followed by the analysis on the correlation of high/low PSMA5 expression with the survival of HCC patients.

### 2.2 Cell culture and transfection

Based on previous reports, the highly metastatic human HCC cell line (MHCC97‐H) purchased from Zhong Qiao Xin Zhou Biotechnology (ZQ0020) was utilized in our study.^30^ MHCC97‐H cells were grown in high‐glucose DMEM (12430047; Thermo Fisher Scientific) supplemented with 10% fetal bovine serum (FBS; GC60166; GLPBIO) and 1% penicillin−streptomycin (15140122; Thermo Fisher Scientific). Human macrophages (THP‐1 cells), which were used for exosome internalization and HCC cell coculture, were obtained from American Type Culture Collection (ATCC; TIB‐202), and maintained in RPMI‐1640 medium (30‐2001; ATCC) added with 0.05 mM 2‐mercaptoethanol (21985023; Thermo Fisher Scientific) and 10% FBS. Cell culture was carried out at 37°C in a humid atmosphere with 10% CO_2_.

PSMA5 was knocked down in MHCC97‐H cells via transfection with short hairpin RNA against PSMA5 (shPSMA5; shPSMA5‐1/−2), which was synthesized by OriGene. pRS shRNA vectors (TR20003; OriGene) served as the negative control (shNC) of shPSMA5.

MHCC97‐H cells were transfected with shPSMA5 or shNC by employing Lipofectamine 3000 transfection reagent (L3000015; Thermo Fisher Scientific). In brief, MHCC97‐H cells (1 × 10^4^ cells/well) were inoculated in 96‐well plates and cultured until reaching 80% confluence. The plasmids and Lipofectamine 3000 transfection reagent were incubated together with both Opti‐MEM and P3000 reagent at 37°C for 15 min to generate gene−lipid complexes. Afterwards, cells were treated with the complexes for 48 h, followed by analysis of the transfection efficiency via quantitative reverse transcription polymerase chain reaction (qRT‐PCR).

### 2.3 Exosome isolation and determination

Exosomes were isolated from the supernatant of MHCC97‐H cells via ultracentrifugation, as previously described.^31^ Briefly, transfected/non‐transfected MHCC97‐H cells were cultured in their medium to be 80% confluent and then transferred into serum‐free DMEM. Following 2 days of culture, the cells were centrifuged at 300×*g* for 15 min, 2000×*g* for 15 min, and 10,000×*g* for 30 min. The supernatant was according obtained and later filtered using a 0.2 μm polyvinylidene fluoride (PVDF) filter (LC2002; Thermo Fisher Scientific). Exosomes were isolated from the harvested supernatant via ultracentrifugation at 120,000×*g* for 70 min twice and identified using Western blot analysis.

### 2.4 Exosome labeling, treatment, and tracking

The internalization of exosomes by THP‐1 cells was visualized via fluorescence labeling and tracking. First, a PKH67 Fluorescent Cell Ligation kit (PKH67GL; Sigma‐Aldrich) was employed to label exosomes. In short, exosomes were treated with Trypsin plus EDTA (59417C; Sigma‐Aldrich), washed with serum‐free DMEM and resuspended in Diluent C. Then, the exosomes were incubated with 2 × PKH Dye Solution at room temperature for 5 min. After being washed by 0.5% bovine serum albumin (BSA; P0007; Beyotime)/phosphate‐buffered saline (PBS; C0221A; Beyotime) to remove excess dye, the labeled exosomes underwent ultracentrifugation at 100,000×*g* for 1 h at 4°C to remove residual dye. Second, the in accordance with a previous report, exosomes at a concentration of 10 µg/mL were incubated with THP‐1 cells at 37°C for 8 h away from light, therefore their internalization into the cells was completed.^32^ THP‐1 cells treated with PBS (C0221A; Beyotime) were adopted as the negative control. Third, the exosomes were tracked in THP‐1 cells. THP‐1 cells treated with exosomes or PBS were seeded in 96‐well plates, and then fixed in 4% paraformaldehyde (V900894; Sigma‐Aldrich) for 10 min, followed by 20 min permeabilization using 0.1% Triton X‐100 (A110694; Sangon). Red Fluorescent Phalloidin Conjugate solution (ab112127; Abcam) was adopted to stain the treated cells at room temperature for 60 min to visualize F‐actin which resides in cytoskeleton. Cell nuclei were counterstained with DAPI (C0065; Solarbio) at room temperature for 5 min. Finally, fluorescent signals were examined by a laser scanning confocal microscope (PCM 2000; Nikon) under ×400 magnification.

### 2.5 QRT‐PCR

Trizol reagent (15596026; Thermo Fisher Scientific) was applied to extract total RNA from transfected/non‐transfected MHCC97‐H cells and treated/nontreated THP‐1 cells. The obtained total RNA was quantified using NanoDorp (701‐058111; Thermo Fisher Scientific). Next, reverse transcription was carried out using reverse transcription kits (K1622; Yaanda Biotechnology) to produce cDNA, followed by qPCR on a detection system (CFX96; Bio‐Rad Laboratories) with the help of Eastep qPCR Master Mix (LS2062; Promega). The thermocycling parameters were set as: 95°C for 10 min, and 40 cycles of 95°C for 15 s and 60°C for 1 min. Glyceraldehyde 3‐phosphate dehydrogenase (GAPDH) was utilized as an internal control. Relative gene expressions were normalized to GAPDH and calculated via the $2^{‐\Delta \Delta C_{t}}$method.^33^ The primers used were shown in Table 1.

**Table 1:** Primers used in quantitative reverse transcription polymerase chain reaction for related genes.

Genes	Species	Forward	Reverse
PSMA5	Human	5′‐TGCCATGAGTGGGCTAATTG‐3′	5′‐GGCACCTGGATCTGCATCTT‐3′
IL‐10	Human	5′‐GACTTTAAGGGTTACCTGGGTTG‐3′	5′‐TCACATGCGCCTTGATGTCTG‐3′
TGF‐ß	Human	5′‐GGCCAGATCCTGTCCAAGC‐3′	5′‐GTGGGTTTCCACCATTAGCAC‐3′
GAPDH	Human	5′‐GGAGCGAGATCCCTCCAAAAT‐3′	5′‐GGCTGTTGTCATACTTCTCATGG‐3′

### 2.6 Enzyme‐linked immunosorbent assay (ELISA)

The levels of IL‐10 and IL‐12 in the supernatant of THP‐1 cells treated with/without MHCC97‐H cell‐derived exosomes or PBS were determined using ELISA kits (ab100549 and ab46035; Abcam). Briefly, centrifugation at 1000×*g* for 5 min was performed at 4°C to collect the supernatant, 100 µL of which was added into precoated 96‐well plates. After incubation at room temperature for 2.5 h, cells were subjected to rinse four times with wash solution, and 60 min further incubation at room temperature with addition of 100 µL Biotin‐labeled antibodies. The treated cells underwent wash gain and treatment with 100 µL horseradish peroxidase (HRP)‐conjugated streptavidin at 37°C for 45 min. A 30 min color development was performed on the cells with TMB substrate solution in the dark after rinsing. Stop solution was later added to stop reaction. Lastly, the color intensity was measured at a wavelength of 450 nm by a microplate reader (PHERAstar FSX; BMG LABTECH).

### 2.7 Cell coculture

For cell coculture, THP‐1 cells treated with transfected/non‐transfected MHCC97‐H cell‐derived exosomes or PBS or not were resuspended in their medium to reach a density of 1 × 10^5^ cells/mL. Thereafter, 1.4 mL of the suspension was poured into the upper compartment of a Transwell chamber (3450; Corning Inc.) with a 0.4‐μm‐pore‐size polycarbonate filter membrane inserted, while the lower compartment was added with 2.5 mL of MHCC97‐H cell suspension (prepared as indicated above). Later, the whole chamber was incubated at 37°C for 24 h.^32^

### 2.8 Wound healing assay

MHCC97‐H cells transfected with shNC/shPSMA5‐1/−2 or MHCC97‐H cells cocultured with THP‐1 cells treated with/without transfected/non‐transfected MHCC97‐H cell‐derived exosomes or PBS were inoculated into six‐well plates at a density of 2 × 10^4^ cells/well, and grown in serum‐free media to form monolayers of 95% confluence. The monolayers were scraped by a sterile pipette tip to create a straight line, and the unattached cells were gently removed by washing with PBS, after which cell incubation was conducted. Control group acted as a blank control and PBS group functioned as a reagent control, while shNC group served as the negative control of shPSMA5‐1 and shPSMA5‐2 groups. Post 0 and 48 h of incubation, the line was captured under ×100 magnification via an inverted microscope (TS100; Nikon).

### 2.9 Transwell assay

A Transwell chamber (3428; Corning Inc.) with 8‐μm‐pore‐size polycarbonate filter membranes was utilized to evaluate the invasive capacity of MHCC97‐H cells. Following with shNC/shPSMA5‐1/−2 or coculture with THP‐1 cells treated with/without transfected/non‐transfected MHCC97‐H cell‐derived exosomes or PBS, MHCC97‐H cells were resuspended with serum‐free DMEM to reach a density of 1 × 10^5^ cells/mL. Control group acted as a blank control and PBS group functioned as a reagent control, while shNC group served as the negative control of shPSMA5‐1 and shPSMA5‐2 groups. The upper compartment was precovered by Matrigel (356234; 50 μL; 1 mg/mL; Corning Inc.), and added with 200 μL of the cell suspension. The culture medium containing 20% FBS was loaded in the lower compartment. The cell incubation was conducted at 37°C for 48 h. After that, invading cells that reached the lower chamber were fixed in 4% paraformaldehyde for 10 min. The fixed cells were stained with 0.5% crystal violet (C0121; Beyotime) for 15 min, and stained cells were observed via the inverted microscope under ×250 magnification.

### 2.10 Murine xenograft assay

All animal experiments were carried out in line with the guidelines of the National Institutes of Health on Animal Care and Use, and authorized by the Ethics Committee of the animal laboratory of Ningbo University (approval number: 2020‐268). BALB/c nude mice (*n* = 30) aged 6 weeks were used in this study, and hypodermically injected with the suspension (100 μL, 5 × 10^5^ cells/site) of non‐cocultured/cocultured MHCC97‐H cells at the fat pad.^34^ Therein, the non‐cocultured MHCC97‐H cells stood for normally cultured MHCC97‐H cells, and the cocultured MHCC97‐H cells represented that MHCC97‐H cells had been cocultured with PBS‐treated THP‐1 cells or THP‐1 cells treated with exosomes isolated from shNC/shPSMA5‐transfected MHCC97‐H cells. MHCC97‐H cells undergoing above treatments were injected into mice of different groups (*n* = 6 per group). The mice injected with non‐cocultured MHCC97‐H cells served as the blank control, and those receiving injection of MHCC97‐H cells that had cocultured with PBS‐treated THP‐1 cells acted as the negative control. Tumors were allowed to grow for 21 days, and the volume of the tumors was measured at Day(s) 0, 3, 7, 14, and 21 following MHCC97‐H cell injection. Tumor volume was calculated as follows: tumor volume (mm^3^) = width (mm)^2^ × length (mm) × 0.5. At Day 21, the mice succumbed to cervical dislocation under anesthetization using 1% pentobarbital sodium (P010; 50 mg/kg; Sigma‐Aldrich), and then the tumors were isolated and weighed.

### 2.11 Immunohistochemistry assay

Following fixation with 4% paraformaldehyde for 24 h, murine tumors were sequentially treated with gradient ethanol and xylene (A530011; Sangon), followed by paraffinization (A606115; Sangon). The paraffinized tumors were cut into 4‐µm‐thick slices, which were then deparaffinized and rehydrated. The slices were immersed in 3% H_2_O_2_ (88597; Sigma‐Aldrich) for 15 min to quench endogenous peroxidase. Then, the slices were microwaved with antigen retrieval solution (P0088; Beyotime) for 5 min, after which 0.1% Triton X‐100 (A110694; Sangon) was used to permeabilize the slices for 10 min. Later, the slices were blocked in 10% BSA (A602449; Sangon) at room temperature for 1 h, and incubated with an antibody against the M2 surface maker CD206 (MA5‐16871; Thermo Fisher Scientific) at 4°C overnight. After that, the HRP‐conjugated goat anti‐Rat IgG secondary antibody (31470; Thermo Fisher Scientific) was employed for further incubation, followed by color development using the Pierce DAB substrate kit (34002; Thermo Fisher Scientific). In addition, hematoxylin (H8070; Solarbio) was used for 5 min of counterstaining. The mice injected with non‐cocultured MHCC97‐H cells served as the blank control, and those undergoing injection of MHCC97‐H cells that had cocultured with PBS‐treated THP‐1 cells acted as the negative control. M2 polarization with CD206 activity was observed by the inverted microscope under ×100 magnification.

### 2.12 Western blot analysis

Total proteins were extracted from exosomes, THP‐1 cells treated with or without exosomes/PBS and murine tumors under the assistance of RIPA Buffer (89900; Thermo Fisher Scientific) containing phosphatase and protease inhibitor mixture (PPC1010; Sigma‐Aldrich). Next, the protein concentration was quantified using a BCA kit (A53227; Thermo Fisher Scientific). SDS‐PAGE gel (89888; Thermo Fisher Scientific) was utilized for total protein (45 µg) separation. The separated proteins were transferred via wet electrophoretic transfer onto a PVDF membrane (P2438; Sigma‐Aldrich). The membrane was later blocked in 5% BSA dissolved in TBST (T9039; Sigma‐Aldrich) at room temperature for 1 h. Thereafter, primary antibodies, including those against extracellular vesicle marker CD63 (ab134045, 50 kDa, 1:1000, Abcam), tumor susceptibility gene 101 (TSG101) (ab125011, 45 kDa, 1:1000, Abcam), extracellular vesicle marker CD9 (ab236630, 25 kDa, 1:1000, Abcam), PSMA5 (ab109387, 26 kDa, 1:1000, Abcam), phosphorylated (p)‐JAK2 (ab32101, 120 kDa, 1:1000, Abcam), JAK2 (ab108596, 130 kDa, 1:1000, Abcam), p‐STAT3 (ab76315, 88 kDa, 1:2000, Abcam), STAT3 (ab68153, 88 kDa, 1:2000, Abcam), and the internal control GAPDH (ab9485, 37 kDa, 1:2000, Abcam), were used to probe the membrane at 4°C overnight. Subsequently, the membrane was washed with TBST, and incubated with HRP‐conjugated goat anti‐rabbit IgG secondary antibody (ab205718; Abcam) at room temperature for 1 h. Protein signals were developed on an imaging system (LAS‐3000; Fujifilm) with enhanced chemiluminescence reagent (WP20005; Thermo Fisher Scientific), followed by densitometrical analysis using Fusion FX Spectra software (7.0 version; Vilber).

### 2.13 Statistical analysis

All data from experiments repeated in triplicate were statistically analyzed by GraphPad prism (version 8.0; GraphPad Software Inc.) and presented as mean ± standard deviation (SD). One‐way analysis of variance (ANOVA) was conducted to compare data among multiple groups. *p* < .05 was regarded as statistical significance.

## 3 RESULTS

### 3.1 High PSMA5 expression was observed in LIHC tissues and associated with reduced survival rate of LIHC patients

**Figure 1:**
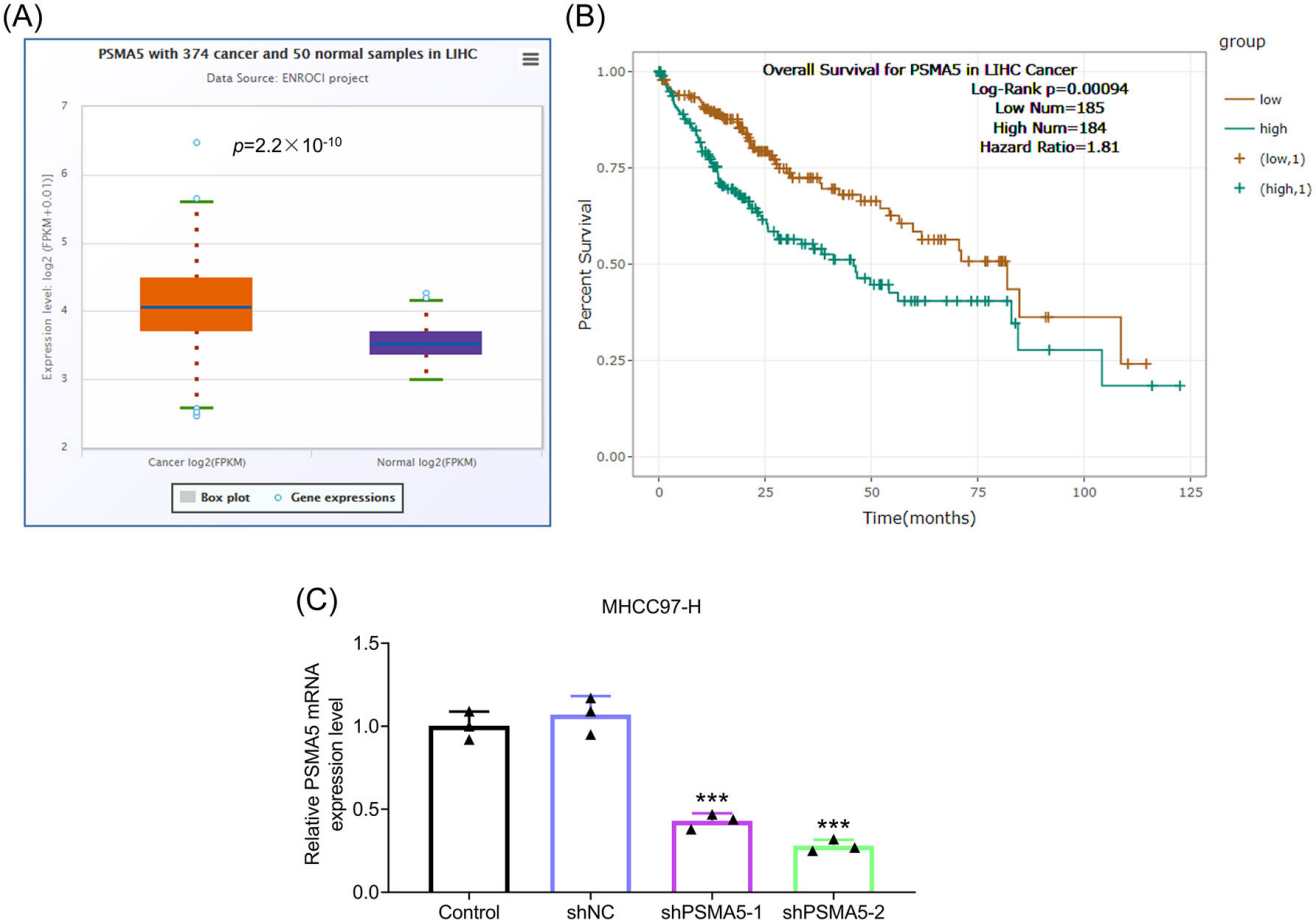
High PSMA5 expression was observed in LIHC tissues and associated with the reduced survival rate of LIHC patients. (A, B). StarBase (https://starbase.sysu.edu.cn/starbase2/) was utilized to predict the expression pattern of PSMA5 in LIHC tissues (A), followed by the analysis on the correlation of high/low PSMA5 expression with the survival of patients with HCC (B). (C) The expression of PSMA5 in MHCC97‐H cells transfected with shNC or shPSMA5‐1/−2 was detected by qRT‐PCR, with GAPDH serving as the normalizer. ***p < .001; * versus shNC. GAPDH, glyceraldehyde 3‐phosphate dehydrogenase; HCC, hepatocellular carcinoma; LIHC, liver hepatocellular carcinoma; PSMA5, proteasome subunit alpha 5; qRT‐PCR, quantitative reverse transcription polymerase chain reaction; shNC, short hairpin RNA against negative control; shPSMA5, short hairpin RNA against PSMA5.

As StarBase‐based prediction indicated, PSMA5 was highly expressed in LIHC tissues, relative to that in normal tissues (Figure 1A, *p* < .001). The median of the PSMA5 expression level in patients with LIHC was used as the cut‐off value to dichotomize the patients into those with high/low PSMA5 expression. Through Kaplan−Meier's estimates, high PSMA5 expression was uncovered to be correlated with a shorter survival time of LIHC patients, relative to low PSMA5 expression (Figure 1B, *p* < .001). To decipher the role of PSMA5 in modulating the malignant behaviors of HCC cells, PSMA5 expression manipulation was carried out in MHCC97‐H cells. PSMA5 was knocked down in MHCC97‐H cells after transfection with shPSMA5‐1/−2 (Figure 1C, *p* < .001).

### 3.2 PSMA5 knockdown inhibited the migration and invasion of HCC cells

**Figure 2:**
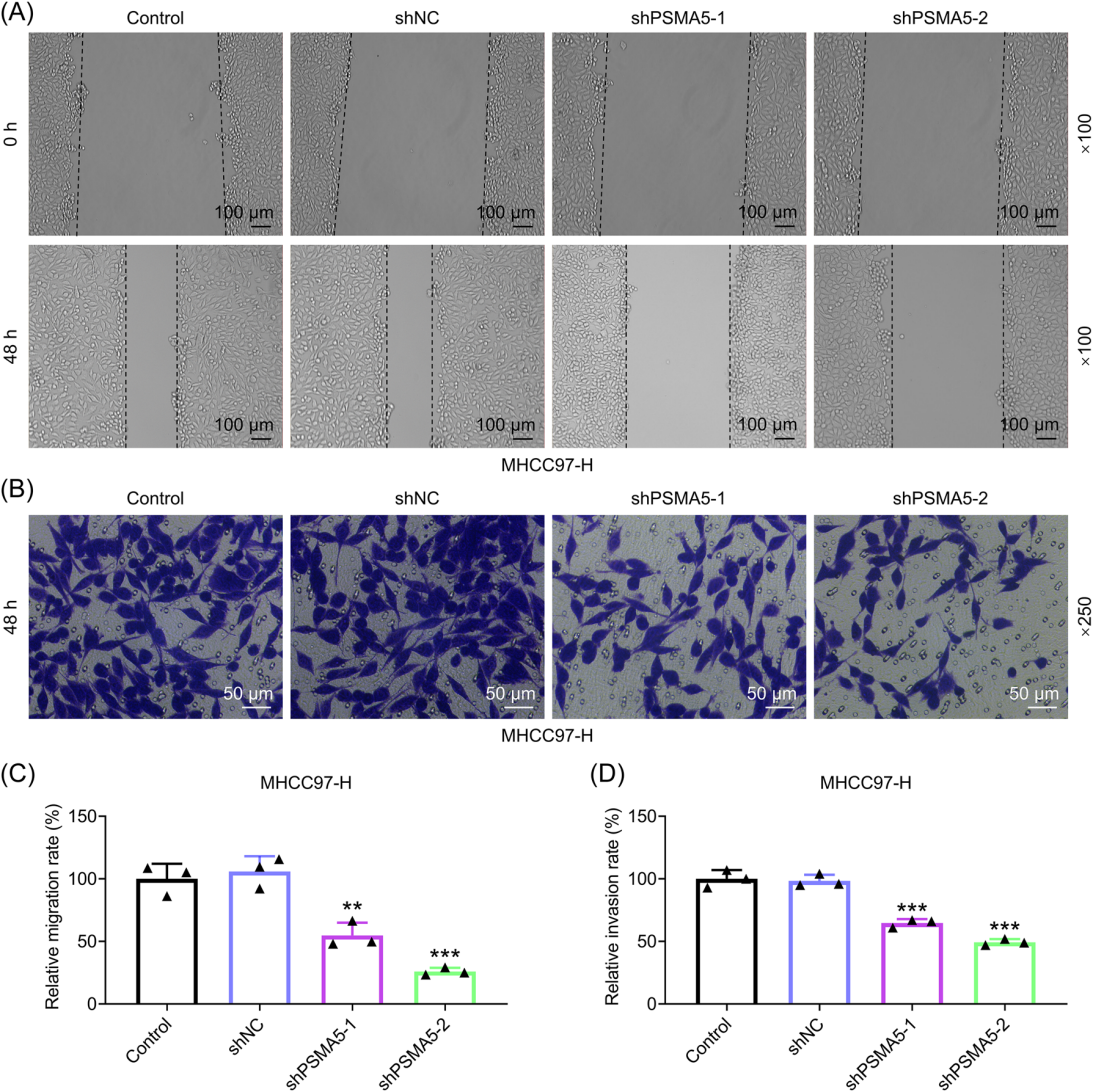
PSMA5 knockdown inhibited the migration and invasion of HCC cells. (A, C) The migration of MHCC97‐H cells transfected with shNC or shPSMA5‐1/−2 was evaluated by wound healing assay (magnification: ×100; scale: 100 µm). (B, D). The invasion of MHCC97‐H cells transfected with shNC or shPSMA5‐1/−2 was evaluated by Transwell assay (magnification: ×250; scale: 50 µm). **p < .01; ***p < .001; * versus shNC. HCC, hepatocellular carcinoma; PSMA5, proteasome subunit alpha 5; shNC, short hairpin RNA against negative control; shPSMA5, short hairpin RNA against PSMA5.

The wound healing assay and Transwell assay were conducted in MHCC97‐H cells transfected with shPSMA5. In comparison to shNC transfection, the transfection of shPSMA5‐1 and shPSMA5‐2 led to a significant reduction in migration speed, as well as a decrease in invasiveness (Figure 2A−D, *p* < .01).

### 3.3 Exosomes isolated from HCC cells could be internalized into macrophages

**Figure 3:**
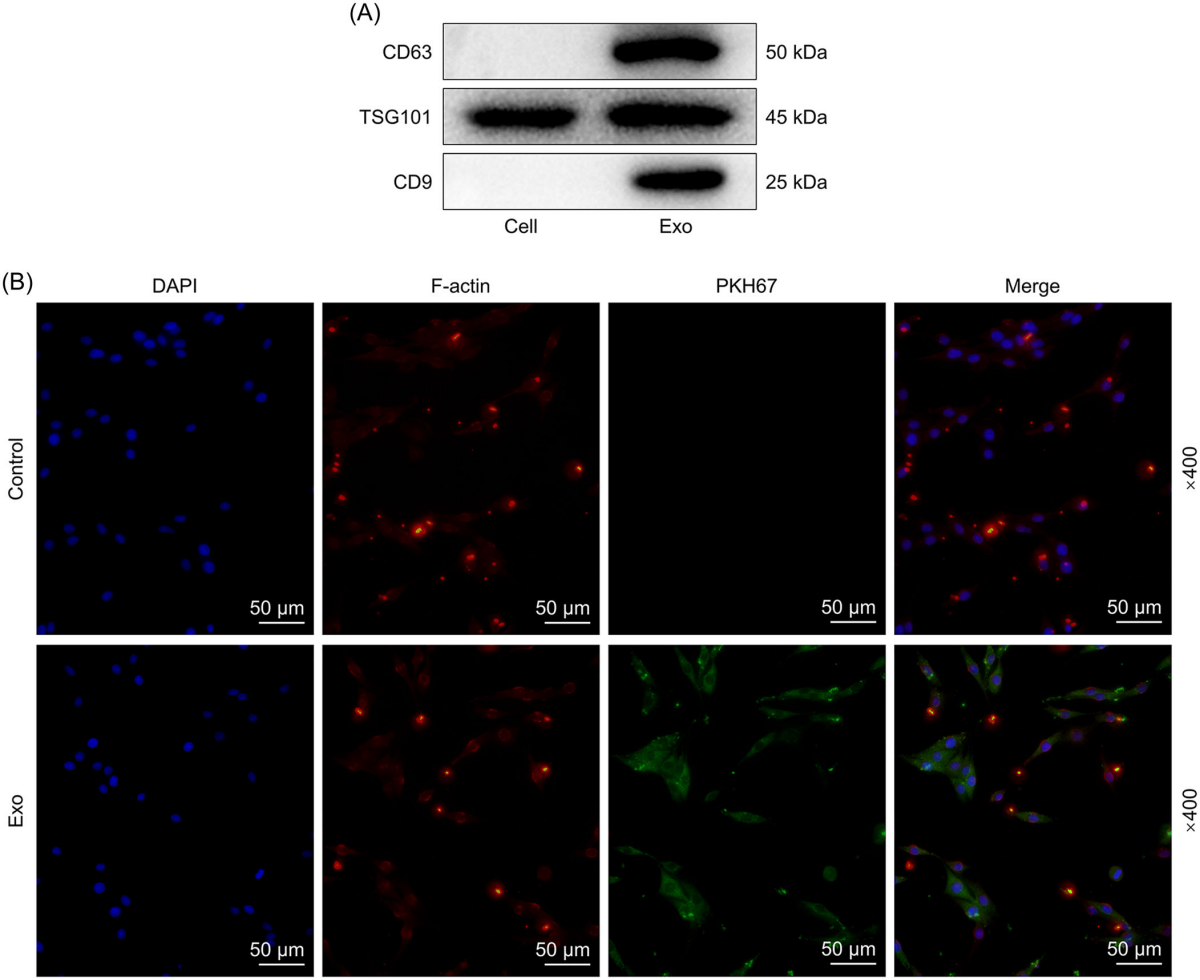
Exosomes isolated from HCC cells could be internalized into macrophages. (A) Exosomes were isolated from MHCC97‐H cells and identified by Western blot analysis, with antibodies against CD63, TSG101, and CD9. (B) The internalization of MHCC97‐H cell‐isolated exosomes into THP‐1 cells was observed after PKH67 labeling of exosomes (magnification: ×400; scale: 50 µm). DAPI, 4′,6‐diamidino‐2‐phenylindole; HCC, hepatocellular carcinoma; PSMA5, proteasome subunit alpha 5; shNC, short hairpin RNA against negative control; shPSMA5, short hairpin RNA against PSMA5; TSG101, tumor susceptibility gene 101.

Exosomes can mediate intercellular communications that modify molecular mechanisms which underlie HCC progression.^15^ As determined by Western blot analysis, exosomes were isolated from MHCC97‐H cells, and identified to show higher levels of CD63, TSG101, and CD9 than the cells (Figure 3A). Infiltration of macrophages into tumors generally heralds the poor prognosis of patients with most cancers.^35^ Fluorescence tracking results illustrated that MHCC97‐H cell‐isolated exosomes could be internalized into macrophages (Figure 3B).

### 3.4 HCC cell‐isolated exosomes facilitated macrophage polarization into M2

**Figure 4:**
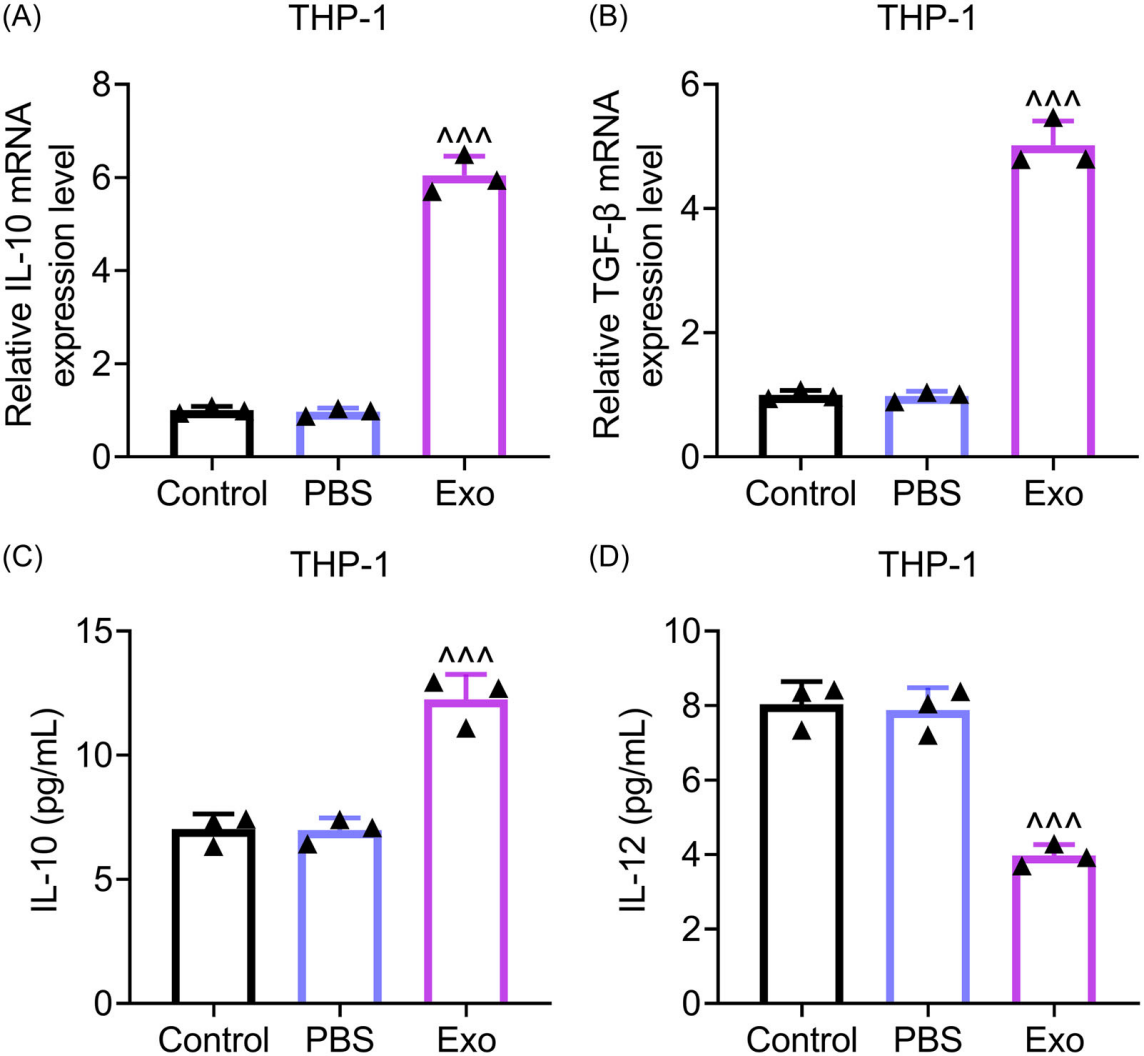
HCC cell‐isolated exosomes facilitated macrophage polarization into M2. (A, B) The expressions of IL‐10 and TGF‐β in THP‐1 cells treated with PBS or MHCC97‐H cell‐isolated exosomes were detected by qRT‐PCR, with GAPDH serving as the normalizer. (C, D). The levels of IL‐10 and IL‐12 in THP‐1 cells treated with PBS or MHCC97‐H cell‐isolated exosomes were determined by ELISA. ^^^^^ *p* < .001; ^^^ versus PBS. ELISA, enzyme‐linked immunosorbent assay; Exo, exosomes; GAPDH, glyceraldehyde 3‐phosphate dehydrogenase; HCC, hepatocellular carcinoma; IL‐10, interleukin‐10; IL‐12, interleukin‐12; PBS, phosphate‐buffered saline; qRT‐PCR, quantitative reverse transcription polymerase chain reaction; TGF‐β, transforming growth factor‐beta.

Through qRT‐PCR analysis, macrophages‐internalizing exosomes were discovered to exhibit increased expressions of IL‐10 and transforming growth factor‐beta (TGF‐β), compared to those with PBS treatment (Figure 4A,B, *p* < .001). The results of ELISA displayed that the level of IL‐10 was increased yet the level of IL‐12 was decreased in macrophages by exosome treatment relative to PBS treatment (Figure 4C,D, *p* < .001).

### 3.5 Knockdown of HCC cell‐secreted exosomal PSMA5 hindered M2 polarization and JAK2/STAT3 pathway activation induced by HCC cell‐secreted exosomes in macrophages

**Figure 5:**
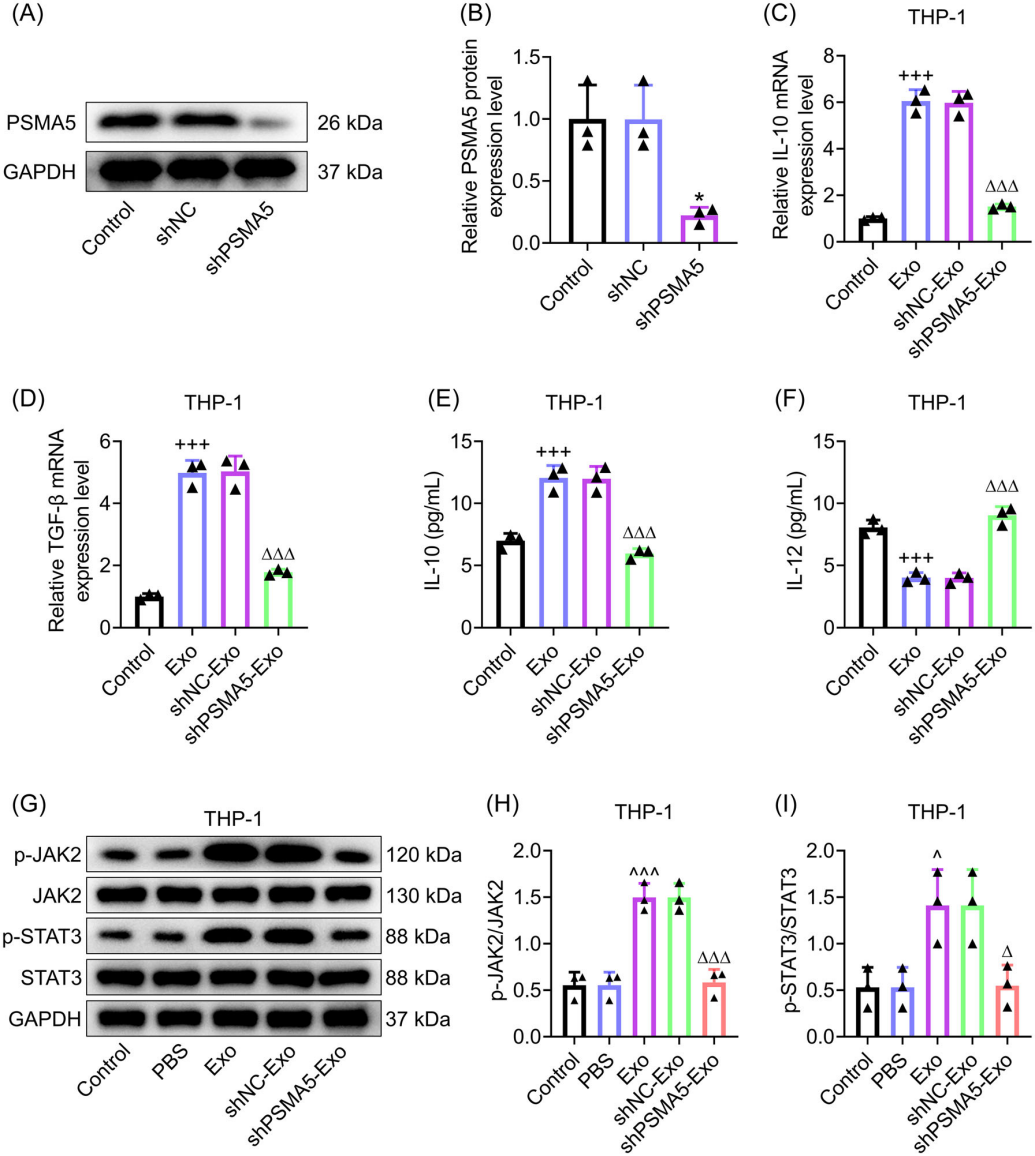
Knockdown of HCC cell‐secreted exosomal PSMA5 hindered M2 polarization and JAK2/STAT3 pathway activation induced by HCC cell‐secreted exosomes in macrophages. (A, B) The expression of PSMA5 in exosomes isolated from MHCC97‐H cells transfected with or without shNC/shPSMA5 was quantified by qRT‐PCR, with GAPDH serving as the normalizer. (C, D) The expressions of IL‐10 and TGF‐β in THP‐1 cells treated with exosomes isolated from MHCC97‐H cells transfected with shNC/shPSMA5 or not were detected by qRT‐PCR, with GAPDH serving as the normalizer. (E, F). The levels of IL‐10 and IL‐12 in the supernatant of THP‐1 cells treated with exosomes isolated from MHCC97‐H cells transfected with shNC/shPSMA5 or not were determined by ELISA. (G−I). The levels of p‐JAK2/JAK2 and p‐STAT3/STAT3 in THP‐1 cells treated with PBS or with exosomes isolated from MHCC97‐H cells transfected with shNC/shPSMA5 or not were measured by Western blot analysis, with GAPDH serving as the normalizer. * *p* or ^^^ *p* or ^△^ *p* < .05; ^+++^ *p* or ^^^^^ *p* or ^△△△^ *p* < .001; * versus shNC; ^+^ versus control; ^^^ versus PBS; ^△^ versus shNC‐Exo. ELISA, enzyme‐linked immunosorbent assay; Exo, exosomes; GAPDH, glyceraldehyde 3‐phosphate dehydrogenase; HCC, hepatocellular carcinoma; IL‐10, interleukin‐10; IL‐12, interleukin‐12; JAK2, Janus kinase 2; M2, M2 macrophages; p‐, phosphorylated‐; PBS, phosphate‐buffered saline; PSMA5, proteasome subunit alpha 5; qRT‐PCR, quantitative reverse transcription polymerase chain reaction; shNC, short hairpin RNA against negative control; shPSMA5, short hairpin RNA against PSMA5; STAT3, signal transducer and activator of transcription 3; TGF‐β, transforming growth factor‐beta.

Subsequently, whether the role of PSMA5 in suppressing HCC cancer progression is attributed to HCC cell‐secreted exosome‐mediated intercellular transport of PSMA5 into macrophages was investigated. ShPSMA5‐2 transfection was singled out for following experiments by dint of its prominent effect on knocking down PSMA5. Exosomes isolated from MHCC97‐H cells transfected with shPSMA5 had a decreased protein expression of PSMA5, when contrasted with those isolated from shNC‐transfected MHCC97‐H cells (Figure 5A,B, *p* < .05). Moreover, in macrophages, exosomes isolated from shPSMA5‐transfected MHCC97‐H cells decreased the expressions of IL‐10 and TGF‐β, relative to exosomes isolated from shNC‐transfected MHCC97‐H cells (Figure 5C,D, *p* < .001). The elevation of IL‐10 level and the lowering of IL‐12 level were observed in macrophages with coculture of exosomes isolated from shNC‐transfected MHCC97‐H cells, but these trends were weakened in macrophages undergoing coculture of exosomes isolated from shPSMA5‐transfected MHCC97‐H cells (Figure 5E,F, *p* < .001). Additionally, in macrophages following treatment with MHCC97‐H cell‐isolated exosomes, the levels of p‐JAK2/JAK2 and p‐STAT3/STAT3 were augmented (Figure 5G−I, *p* < .05), which however was repressed by underexpressing PSMA5 in these MHCC97‐H cell‐isolated exosomes (Figure 5G−I, *p* < .05).

### 3.6 Knockdown of HCC cell‐secreted exosomal PSMA5 inhibited HCC cell migration and invasion induced by HCC cell‐secreted exosomes

**Figure 6:**
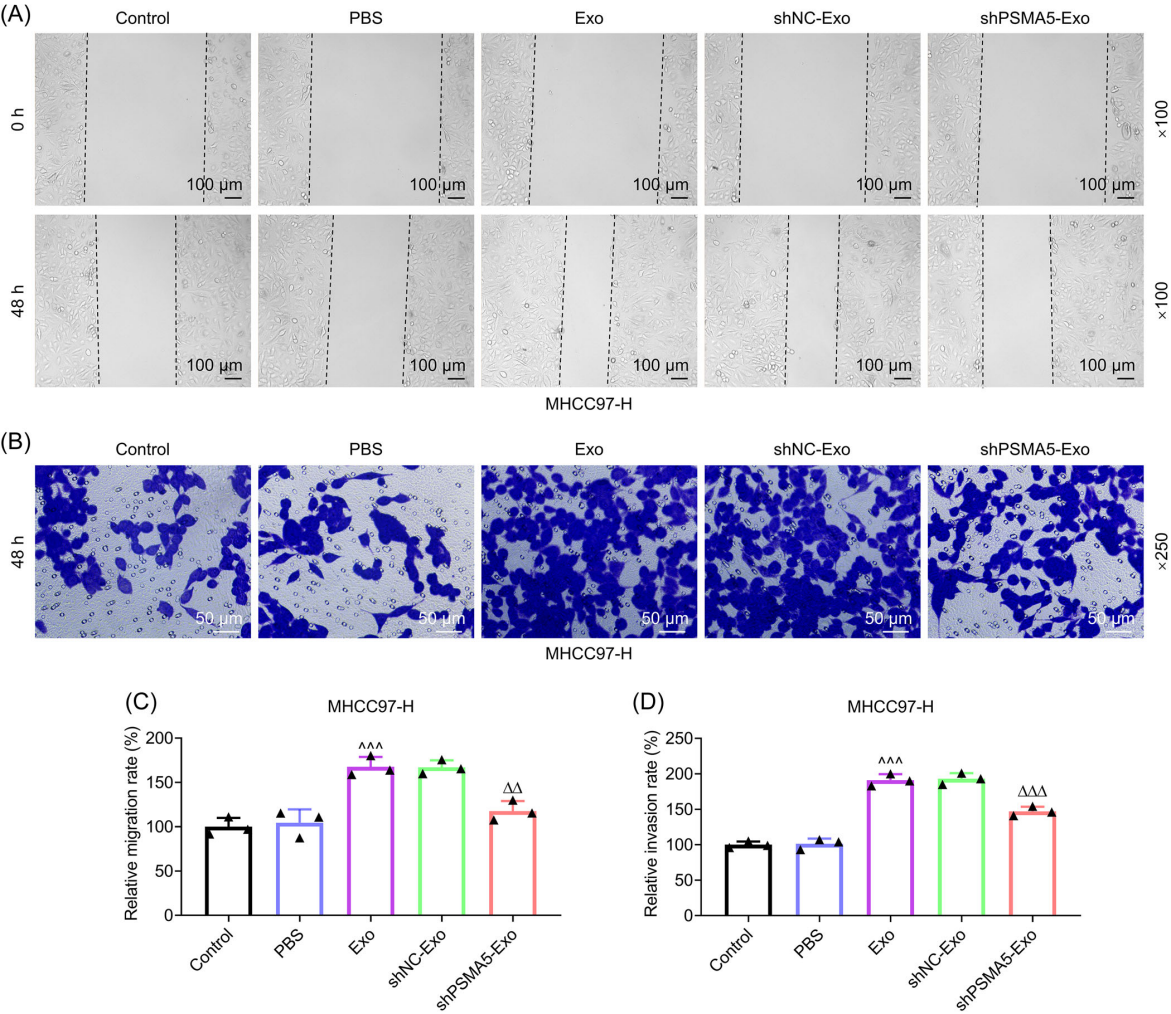
Knockdown of HCC cell‐secreted exosomal PSMA5 inhibited HCC migration and invasion induced by HCC cell‐secreted exosomes. (A, C) The migration of MHCC97‐H cells cocultured with THP‐1 cells under treatment of PBS or exosomes isolated from MHCC97‐H cells transfected with shNC/shPSMA5 or not was evaluated by wound healing assay (magnification: ×100; scale: 100 µm). (B, D) The invasion of MHCC97‐H cells cocultured with THP‐1 cells under treatment of PBS or exosomes isolated from MHCC97‐H cells transfected with shNC/shPSMA5 or not was evaluated by Transwell assay (magnification: ×250; scale: 50 µm). ^△△^ *p* < .01; ^^^^^ *p* or ^△△△^ *p* < .001; ^^^ versus PBS; ^△^ versus shNC‐Exo. Exo, exosomes; HCC, hepatocellular carcinoma; PBS, phosphate‐buffered saline; PSMA5, proteasome subunit alpha 5; shNC, short hairpin RNA against negative control; shPSMA5, short hairpin RNA against PSMA5.

Furthermore, macrophages treated with MHCC97‐H cell‐derived exosomes, as contrasted with macrophages treated with PBS, enhanced the migratory and invasive capacities of MHCC97‐H cells (Figure 6A−D, *p* < .001), but this enhancement was attenuated when these macrophages were treated with exosomes isolated from PSMA5‐silenced MHCC97‐H cells (Figure 6A−D, *p* < .01).

### 3.7 Knockdown of HCC cell‐secreted exosomal PSMA5 reversed the promotion of HCC tumorigenesis induced by macrophages undergoing treatment of HCC cell‐secreted exosomes

**Figure 7:**
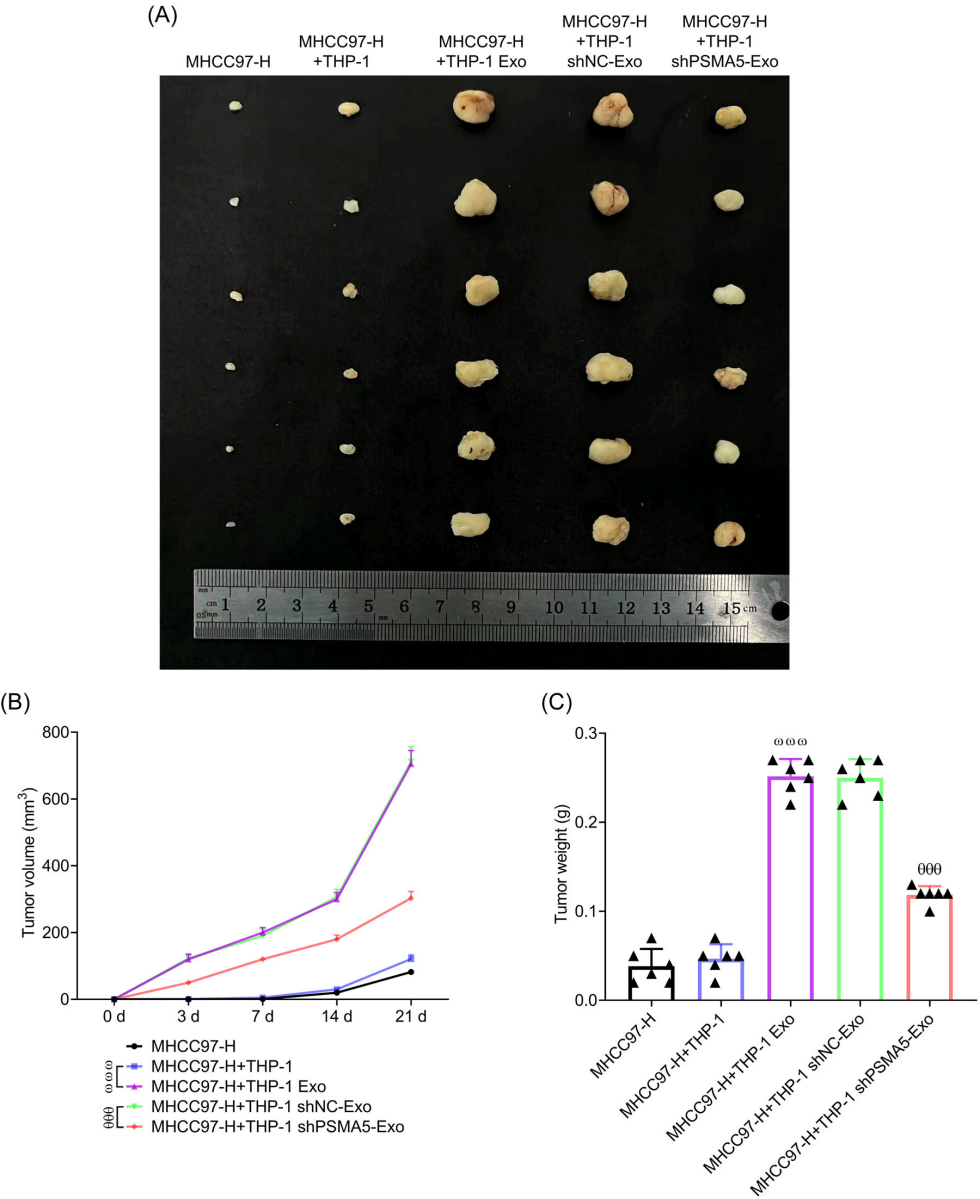
Knockdown of HCC cell‐secreted exosomal PSMA5 reversed the promotion of HCC tumorigenesis induced by macrophages undergoing treatment of HCC cell‐secreted exosomes. (A−C) Murine xenograft assay was carried out. BALB/c nude mice were hypodermically injected with cell suspension (100 μL, 5 × 10^5^ cells/site) of MHCC97‐H cells which had been cocultured for 24 h with THP‐1 cells undergoing treatment of PBS or exosomes isolated from shNC/shPSMA5‐transfected MHCC97‐H cells or not at the fat pad of the mice. (B, C). The volume of the tumors was measured at Days 0, 3, 7, 14, and 21 following MHCC97‐H cell injection (B). At Day 21 following MHCC97‐H cell injection, the tumors were weighed (C). ^ωωω^ *p* or ^θθθ^ *p* < .001; ^ω^ versus MHCC97‐H + THP‐1; ^θ^ versus MHCC97‐H + THP‐1 shNC‐Exo. Exo, exosomes; HCC, hepatocellular carcinoma; PBS, phosphate‐buffered saline; PSMA5, proteasome subunit alpha 5; shNC, short hairpin RNA against negative control; shPSMA5, short hairpin RNA against PSMA5.

The results of xenograft assay revealed that at Day(s) 0, 3, 7, 14, and 21 (weight measurement only at Day 21), the volume and weight of the formed tumors in mice were increased obviously in MHCC97‐H + THP‐1 Exo, MHCC97‐H + THP‐1 shNC‐Exo, and MHCC97‐H + THP‐1 shPSMA5‐Exo groups (Figure 7A−C, *p* < .001). Further, at Day(s) 0, 3, 7, 14, and 21 (weight measurement only at Day 21), the tumor volume was smaller and the tumor weight was lighter in MHCC97‐H + THP‐1 shPSMA5‐Exo group than those in MHCC97‐H + THP‐1 shNC‐Exo group (Figure 7A−C, *p* < .001).

### 3.8 Knockdown of HCC cell‐secreted exosomal PSMA5 reversed M2 infiltration and JAK2/STAT3 pathway activation induced by macrophages treated with HCC cell‐secreted exosomes in HCC tumors

**Figure 8:**
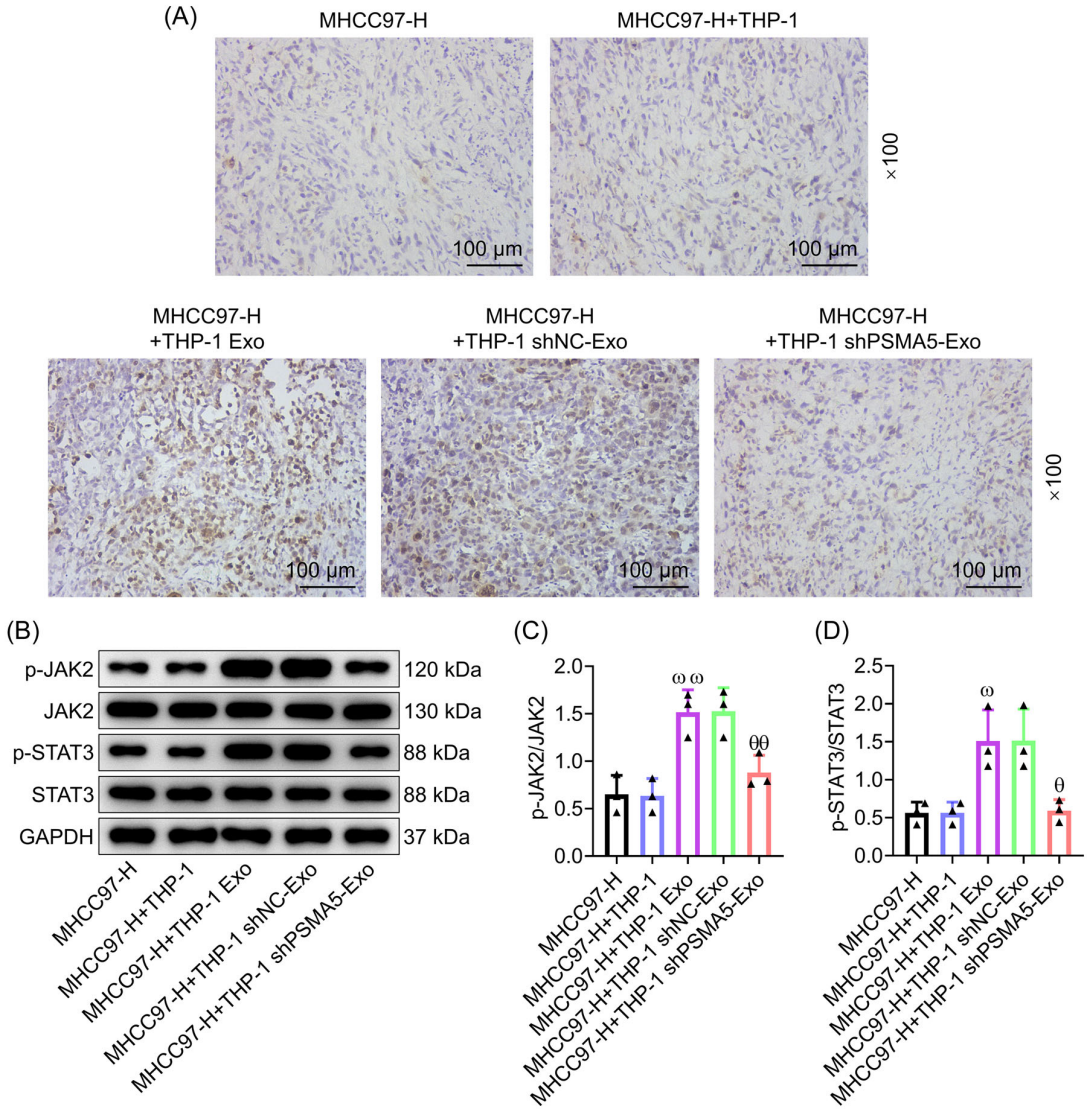
Knockdown of HCC cell‐secreted exosomal PSMA5 reversed M2 infiltration and JAK2/STAT3 pathway activation by macrophages treated with HCC cell‐secreted exosomes in HCC tumors. (A−D) Murine xenograft assay was carried out. BALB/c nude mice were hypodermically injected with cell suspension (100 μL, 5 × 10^5^ cells/site) of MHCC97‐H cells which had been cocultured for 24 h with THP‐1 cells undergoing treatment of PBS or exosomes isolated from shNC/shPSMA5‐transfected MHCC97‐H cells or not at the fat pad of the mice. (A) M2 polarization in the tumors was assessed by immunohistochemistry using CD206 antibody (magnification: ×100; scale: 100 µm). (B−D) The levels of p‐JAK2/JAK2 and p‐STAT3/STAT3 in the tumors were detected by Western blot analysis, with GAPDH serving as the normalizer. ^ω^ *p* or ^θ^ *p* < .05; ^ωω^ *p* or ^θθ^ *p* < .01; ^ω^ versus MHCC97‐H + THP‐1; ^θ^ versus MHCC97‐H + THP‐1 shNC‐Exo. Exo, exosomes; GAPDH, glyceraldehyde 3‐phosphate dehydrogenase; HCC, hepatocellular carcinoma; JAK2, Janus kinase 2; M2, M2 macrophages; p‐, phosphorylated‐; PBS, phosphate‐buffered saline; PSMA5, proteasome subunit alpha 5; shNC, short hairpin RNA against negative control; shPSMA5, short hairpin RNA against PSMA5; STAT3, signal transducer and activator of transcription 3.

Meanwhile, the results of immunohistochemistry assay illustrated that the expression of CD206 was increased in the MHCC97‐H + THP‐1 Exo group as compared with that in MHCC97‐H + THP‐1 group (Figure 8A). Low expression of CD206 was observed in MHCC97‐H + THP‐1 shPSMA5‐Exo group relative to MHCC97‐H + THP‐1 shNC‐Exo group (Figure 8A). Besides, the levels of p‐JAK2/JAK2 and p‐STAT3/STAT3 were increased in the MHCC97‐H + THP‐1 Exo group as compared with those in MHCC97‐H + THP‐1 group (Figure 8B−D, *p* < .05). The diminished levels of p‐JAK2/JAK2 and p‐STAT3/STAT3 were observed in MHCC97‐H + THP‐1 shPSMA5‐Exo group relative to MHCC97‐H + THP‐1 shNC‐Exo group (Figure 8B−D, *p* < .05).

## 4 DISCUSSION

HCC is one of the most frequent malignancies, with a low survival rate round the world.^3^ In United States, patients with HCC presented an average 5‐year survival rate of 19.6%, and this rate even declines to be as low as 2.5% for those with advanced, metastatic disease,^1^ highlighting the importance of preventing the progression of HCC. M2‐phenotype TAMs that abundantly exist in the TME exert a pro‐tumor effect.^36^ The present study discovered that knockdown of HCC cell‐secreted exosomal PSMA5 suppressed HCC cancer progression by hindering M2 macrophage polarization.

Although PSMA5 has been found to be lowly expressed in gliomas,^37^ in many other tumors such as LUAD and prostate cancer, it shows an upregulated expression level.^24^, ^25^ High PSMA5 expression is linked to the poor prognosis of patients with prostate cancer.^24^ Furthermore, it is demonstrated that PSMA5 silencing inhibits proliferation, migration, and invasion and induces apoptosis of prostate cancer and LUAD cells, while increasing the sensitivity of the cells to bortezomib or cisplatin,^24^, ^25^ suggesting that PSMA5 acts as an oncogenic protein in these two types of cancers. In our study, bioinformatics analysis predicted that highly expressed PSMA5 also existed in LIHC and was associated with the reduced survival rate of LIHC patients. In addition to this, consistent with the findings that PSMA5 plays an oncogenic role in LUAD and prostate cancer, our data showed that knockdown of PSMA5 resulted in inhibited HCC cell migration and invasion.

Exosomes, which are produced actively in cancer cells, can be distributed in all body fluids and then releases cargo obtained from the cancer cells to the neighboring cells, thus assisting major cancer hallmarks in sustaining growth and boosting metastasis while constructing ways to evade immune responses.^15^, ^38^ TAMs are one kind of the neighboring immune cells that experience exosome‐mediated communication with cancer cells to implement immune evasion.^39^ After being recruited into the TME of HCC, in response to the TME signals, TAMs undertake polarization into M2.^8^, ^40^, ^41^ M2 shows a distinct secretory profile, which leads to the low level of IL‐12 and high levels of TGF‐β and IL‐10, thereby promoting the anti‐inflammatory/protumorigenic responses.^42^, ^43^ Previously, HCC cell‐derived exosomes have been proven to increase the immuno‐suppressive M2 phenotype of macrophages.^44^ In line with the findings above, the results in the present study displayed that HCC cell‐derived exosomes were internalized into macrophages, and led to the upregulation of TGF‐β and IL‐10 as well as downregulation of IL‐12, which highlighted the role of HCC cell‐derived exosomes in modulating the immune response and inflammation. Understanding the mechanisms by which exosomes affect macrophage behaviors may provide novel insights for developing novel therapeutic strategies targeting immune evasion in HCC.

Specific proteins transferred by HCC cell‐derived exosomes to macrophages infiltrating the TME promote HCC immune escape,^18^ and HCC cell‐secreted exosomal miR‐452‐5p accelerates HCC progression through inducing M2 polarization.^19^ PSMA5 has been reported as a functional factor, whose expression is upregulated in highly metastatic HCC cell‐derived exosomes.^22^ Moreover, PSMA5 level is downregulated in TAMs showing M1‐like phenotype.^28^ In addition, the JAK2/STAT3 pathway, which is involved in diversified biological processes including cell proliferation, differentiation, apoptosis, and immune regulation, plays a critical role in mediating tumorigenesis,^45^ and has been uncovered to be repressed along with inhibition of M2 polarization during suppressed gastric cancer progression.^46^ Of note, PSMA5 can activate the JAK2/STAT3 pathway, thus contributing to LUAD progression.^25^ These findings, together with our results that PSMA5 expression was upregulated in HCC, prompted us to make a presumption that PSMA5 in HCC cells was transferred by HCC cell‐derived exosomes into macrophages to drive M2 polarization via activating the JAK2/STAT3 pathway, thereby exerting an oncogenic effect on HCC cells. Conforming to this presumption, in our study, PSMA5 knockdown in HCC cells led to the downregulation of PSMA5 in exosomes derived from these HCC cells, and HCC cell‐derived exosomes induced M2 polarization and JAK2/STAT3 pathway activation in macrophages and enhanced the migratory and invasive capabilities of HCC cells. Furthermore, we revealed that underexpressed PSMA5 in HCC cell‐derived exosomes weakened the effect exerted by HCC cell‐derived exosomes in macrophages as well as the HCC cell‐derived exosome‐conferred ability for macrophages to affect HCC cell migration and invasion. The results suggested that PSMA5 in exosomes plays a crucial role in mediating the communication between HCC cells and macrophages. Specifically, PSMA5 in exosomes can make impacts upon the abilities of HCC cells to migrate and invade, which is realized through M2 polarization of macrophages and activation of the JAK2/STAT3 pathway.

To revalidate our results from in vitro experiments, we performed murine xenograft assay, where similar results were obtained. To be specific, tumor growth, M2 infiltration, and JAK2/STAT3 pathway activation induced by macrophages undergoing treatment of HCC cell‐derived exosomes were all compromised when the macrophages were treated with exosomes derived from HCC cells underexpressing PSMA5.

Our study provides valuable insights into the role of PSMA5 in HCC progression and its therapeutic implications. However, there are still several limitations. For instance, we merely focused on a single HCC cell line, which may not fully represent the complexity of HCC across different patients. Additionally, the results in our study were obtained from in vitro experiments, which may not fully replicate the TME in vivo. Further research using animal models or clinical samples is needed to validate our findings and assess the potential of targeting PSMA5 in HCC treatment.

## 5 CONCLUSION

In conclusion, the present work is the first to report that HCC shows highly expressed PSMA5 and knockdown of PSMA5 suppresses HCC progression. More importantly, this anti‐HCC effect can be achieved by downregulating PSMA5 level in HCC cell‐derived exosomes to hinder M2 polarization. These results offer a potential way of lowering the advanced tumor stages to fulfill the criteria of locoregional treatment from the perspective of targeting M2‐mediated immunosuppression.

## AUTHOR CONTRIBUTIONS

Shujie Xie performed the research. Shujie Xie designed the research study. Xiang Li, Jia Yan, Hua Yu, Shuhuai Chen, and Kana Chen contributed essential reagents or tools. Xiang Li, Jia Yan, Hua Yu, Shuhuai Chen, and Kana Chen analyzed the data. All authors wrote the paper. All authors have read and approved the final manuscript.

## CONFLICT OF INTEREST STATEMENT

The authors declare no conflict of interest.

## Data Availability

All data included in this study are available upon request by contact with the corresponding author.
